# Preventing Cardiotoxicity in Personalized Breast Irradiation

**DOI:** 10.3390/cancers15215153

**Published:** 2023-10-26

**Authors:** Edy Ippolito, Carlo Greco, Maristella Marrocco, Carla Germana Rinaldi, Michele Fiore, Luca Eolo Trodella, Rolando Maria D’Angelillo, Sara Ramella

**Affiliations:** 1 Radiation Oncology, Campus Bio-Medico University Rome, 00128 Rome, Italy; e.ippolito@unicampus.it (E.I.); c.greco@policlinicampus.it (C.G.); ma.marrocco@ausl.latina.it (M.M.); m.fiore@unicampus.it (M.F.); luca.trodella@unicampus.it (L.E.T.); s.ramella@unicampus.it (S.R.); 2 Radiotherapy, Department of Oncoematology, Policlinico Tor Vergata, 00133 Rome, Italy; carlagermana.rinaldi@ptvonline.it; 3 Radiation Oncology, Università degli Studi di Roma Tor Vergata, 00133 Rome, Italy

**Keywords:** DIBH, breast cancer, left descending artery, breast irradiation

## Abstract

**Simple Summary:**

Adjuvant radiotherapy is a standard of care in the treatment of breast cancer patients after surgery, but irradiation of left-sided breast cancer showed a higher incidence of adverse cardiac effects, mainly for left descending artery (LAD) irradiation. The aim of the study was to assess the benefit of a deep inspiration breath hold (DIBH) over a standard irradiation technique. Patients received both standard and DIBH simulation. Data on 394 treatment plans (197 patients) were extracted and analyzed. The LAD dose was significantly reduced in DIBH plans with the maximum and mean dose reduced by 31.7% (mean value 3.5 Gy vs. 4.8 Gy, *p* ≤ 0.001) and 28.1% (mean value 8.2 Gy vs. 12.8 Gy, *p* ≤ 0.001) in DIBH plans compared to FB plans, underlying that patients could suffer less from irradiation cardiotoxicity with this technique.

**Abstract:**

Background: This study aims to assess the benefit of a deep inspiration breath hold (DIBH) over the standard irradiation technique, and eventually to identify anatomical and/or treatment preplanning characteristics correlated with the LAD dose. Methods: Patients with left-sided breast cancer undergoing whole breast radiotherapy with DIBH were analyzed. All patients included in the analysis had plans in DIBH and free-breathing (FB). Receiving operating characteristics (ROC analysis) were used to identify the cut-off point of parameters to predict the LAD maximum dose > 10 Gy and LAD mean dose > 4 Gy, and the areas under the curve (AUCs) were computed. Post-test probability has been performed to evaluate the effect of parameters’ combination. Results: One hundred ninety-seven patients were analyzed. The LAD dose was significantly reduced in DIBH plans with the maximum and mean dose reduced by 31.7% (mean value 3.5 Gy vs. 4.8 Gy, *p* ≤ 0.001) and 28.1% (mean value 8.2 Gy vs. 12.8 Gy, *p* ≤ 0.001) in DIBH plans compared to FB plans. The strongest predictor of the LAD dose (maximum > 10 Gy and mean > 4 Gy) was the minimum distance of LAD from tangent open fields. Other parameters were lung volume and heart volume (LAD Dmax > 10 Gy) and lung volume, heart volume, and breast separation (LAD Dmean > 4 Gy). Conclusion: The dosimetric advantage of DIBH is clear in all patients and DIBH should always be preferred.

## 1. Introduction

Breast cancer is the most common malignancy occurring in women, accounting for 30% of female cancers. In the last several years, the disease treatment has improved due to new surgical techniques, new systemic treatment options, and an increased understanding of the biology of the disease. In multidisciplinary breast cancer management, adjuvant radiotherapy plays an essential role in reducing breast tumor recurrence and improving overall survival. Conservative surgery followed by adjuvant radiotherapy is the standard treatment for early-stage breast cancer. Post-mastectomy radiation therapy (PMRT) use has increased in the last few years, making it a critical component of breast cancer treatment [1,2]. 

Despite the benefits of RT and the improvement in radiation techniques observed over the years, left-sided breast cancer radiotherapy has shown to be related to a higher incidence of several adverse cardiac effects, occurring even many years after treatment. These events can also cause premature mortality which is not cancer-related. Left-sided RT is significantly associated with higher cardiovascular mortality compared with right-sided RT, with an increased risk observed even after ≥15 years of follow-up (RR: 1.23, 95% CI: 1.08–1.41, *p* < 0.001). The increase in cardiovascular deaths appears to be mainly related to heart dose exposure. Incidental heart irradiation can also occur in patients treated with more recent RT techniques, as the heart still receives doses ranging between 1 and 5 Gy [3]. 

The development of these long-term cardiovascular toxicities is not completely understood. It is well known that multiple factors are involved in radiation-induced heart damage. The pathophysiological alterations observed include macrovascular and microvascular endothelial injury which develop after endothelial cell damage. In coronary arteries, RT causes fibrointimal hyperplasia, which leads to thrombus formation and potentially lipid deposition, similar to the typical mechanism of coronary artery disease. Also, myocardial remodeling may occur, as well as oxidative stress and inflammation, mainly as late effects. It is unclear whether free radicals produced by irradiation play a direct role in damaging the myocardium. The radiation-induced heart damage has proved to be mainly related to incidental cardiac dose linearly correlated with the incidence of major coronary events. A relative increase in the rate of major coronary events has been observed with an increase of 7.4% per Gy of incidental heart radiation dose with no dose threshold [4,5]. Incidental heart dose appears to also be related to the risk of post-radiotherapy perfusion defects that can be detected early on functional imaging [6] after the radiotherapy course.

Left descending artery (LAD) irradiation has been increasingly recognized as a relevant mechanism of cardiac damage in preclinical [7] and clinical studies. In preclinical murine models, radiotherapy exposure resulted in associated morphological injury to the vascular endothelium of coronaries resulting in stenosis, decreased density of the smaller diameter coronary vessels, and a decrease in ventricular function [7].

In a clinical setting, left-sided breast cancer patients showed more common myocardial perfusion changes in the LAD distribution region, but not in the left circumflex artery and right coronary artery distribution regions [8]. Particularly, tangential treatment planning resulted in associated short-term SPECT defects in the vascular distribution corresponding to the anatomical heart portion included in the radiation portals. Perfusion defects, occurring 6 months after the radiotherapy course, depended not only on left ventricular dose and LAD dose but also on several clinical factors such as concomitant hormonal treatment and pre-existent hypercholesterolemia [8]. LAD all-grades stenosis measured by angiography was found to be more common in left-sided patients compared to right-sided breast cancer patients, especially in the mid and distal regions. A four- to seven-fold increase in high-grade severe stenosis was recorded in left irradiated breast cancer patients [9]. Moreover, coronary artery calcium (CAC) score measurement performed at a median of 32 months after RT showed higher values in patients with greater heart exposure. Particularly, higher CAC scores were associated with higher values of LAD maximum and mean dose, and with the volume of LAD receiving 40 Gy [10]. Women receiving LAD mean doses between 1 and 5 Gy to the mid portion more often needed later coronary intervention compared to women receiving lower mean doses ranging from 0 to 1 Gy [11].

Nowadays, there are several radiation techniques that allow heart sparing by means of: reduced treatment volume to the tumor bed (partial breast irradiation, PBI); advanced techniques such as intensity modulated radiation therapy (IMRT) or volumetric arc therapy (VMAT); and decreased cardiac exposure to radiation (prone position or deep inspiration breath hold—DIBH). However, not all of them can be routinely used. In fact, PBI can be safely used in clinical settings only in low-risk patients with particular well-defined characteristics such as unifocal small-sized tumors, G1-G2, with estrogen receptor expression. Moreover, the use of modern radiotherapy techniques (IMRT and/or VMAT) can decrease the maximum dose to heart substructures but, on the contrary, may increase low-dose exposure to larger heart portions. Conversely, DIBH increasing the physical separation between the chest wall and the heart during inspiration is able to reduce heart exposure in all patients, and therefore it is considered the gold standard technique for heart sparing [12]. Literature data reports a reduction in mean heart dose ranging from 38% to 59% and a reduction in the mean dose to the left descending artery (LAD) ranging from 31% to 71% with DIBH [13]. 

Several studies have investigated predictors of mean heart dose (MHD) reduction with the use of the breath-hold technique. Most studies focused on the predictive value of anatomic factors [13,14]. However, the mean heart dose alone does not seem to be representative of the LAD dose. In fact, despite low mean heart doses, a large portion of the heart can be exposed to higher doses ranging between 40 and 50. This region often corresponds to the area in which the LAD is located. Therefore, given the location of the LAD close to the tangential fields used in left-sided breast RT, the difference between the LAD dose and MHD can be significant. Indeed, patients receiving an increased MHD may not receive a higher mean LAD dose and vice versa [15].

Based on the above considerations, the aim of this study was to identify in a large dataset of patients the anatomical and/or treatment preplanning characteristics correlated with the LAD dose, in order to evaluate the amount of benefit of DIBH over standard treatment, and eventually guide the selection of patients with left-breast cancer and prevent cardiotoxicity.

## 2. Methods and Material

### 2.1. Ethics

This study was performed under the ethical standards of the institutional and national research committee and with the Helsinki Declaration. No specific ethical approval was required for this retrospective dose analysis. All patients included gave consent for data collection according to the study design requirements and the Fondazione Policlinico Universitario Campus Bio-Medico ethical committee.

### 2.2. Patient Selection

Patients with left-sided breast cancer treated with whole-breast adjuvant radiotherapy (WBRT) and DIBH from 2014 to 2018 were identified from the institutional database. All patients were studied as both standard treatment (free-breathing—FB) and DIBH, and for all patients, two plans were realized at the time of initial treatment: one in FB and one in DIBH. Therefore, we performed a quantitative retrospective analysis of dosimetric parameters from treatment plans in FB and DIBH for each patient.

### 2.3. Simulation, Contouring, and Treatment Planning

All patients were treated supine with both arms raised above the head on a customized breast board. All patients received a training session to establish the individual deep inspiration level and optimize compliance with DIBH. Patients who were not able to hold their breath for at least 20 s and maintain a stable breath-hold were deemed not eligible for the DIBH technique. First, patients underwent a FB CT scan and immediately after a DIBH CT scan in the same position. CT scan was performed from the jugular notch to 5 cm below the lower edge of the mammary gland with a scan interval of 5 mm. RTOG guidelines [16] and the heart atlas published by Feng et al. [17] were followed for target volume and organs at risk (OARs) delineation (heart, LAD, contralateral breast, and ipsilateral lung). The breast clinical target volume CTV was the entire mammary gland. The boost CTV was the surgical bed, defined from the location of the surgical clips in the lumpectomy cavity, with a 1 cm margin. The planning target volumes PTV were obtained by adding a 5 mm margin to each CTV, cropping 4 mm inside the patient skin, and excluding ribs and lung parenchyma. Delineations were manually carried out by a radiation oncologist with at least 5 years’ experience. LAD contours were reviewed independently by 2 physicians. Planning was performed using the Eclipse Treatment Planning System (TPS). The total prescribed dose was 50 Gy in 25 fractions. The optimization objectives for PTV were: 95% of the prescription dose to 95% of the PTV volume; 105% of the prescription dose to less than 5% of the PTV volume. For the OARs, the following constraints were used: heart mean dose < 5 Gy (optimal < 3.5 Gy), left descending artery (LDA) Dmax < 20 Gy (optimal < 15 Gy), LDA Dmean < 10 Gy (optimal < 8 Gy), volume of lung receiving 5 Gy (V5) less than 60%, contralateral breast mean dose < 3 Gy.

A simple tangential plan with wedges and gantry angles optimized to match the divergence of the posterior parts of the beam was realized to avoid contralateral irradiation and to minimize the ipsilateral lung and heart irradiation reducing the portions of lung and heart included in the field. Moreover, in some cases, a forward intensity-modulated radiotherapy (IMRT) employing a field-in-field technique or irregular surface compensator technique was applied too. A Varian RPM™ (Varian Medical Systems, Palo Alto, CA, USA) respiratory gating system was used for the delivery and monitoring of treatment in DIBH.

### 2.4. Anatomical and Treatment Planning Data

LAD dosimetric data were collected from both plans (FB and DIBH) for each patient as a mean LAD dose and maximum LAD dose. Furthermore, the differences in these values (Δ, %) between FB and DIBH plans were also calculated.

Moreover, the following anatomical parameters were obtained and recorded for each patient: lung volume (cc), heart volume (cc), breast separation (cm), and minimum distance from LAD to the treatment field. Breast separation was intended as the largest distance between the medial and lateral border of the mammary gland measured on an axial CT scan. The minimum distance from LAD to the treatment field was the shortest distance measured between LAD and tangent open fields on both axial and sagittal scan planes. This parameter was computed by the same two physicians who independently reviewed LAD contours. If concordance was not achieved, a mean value was obtained.

### 2.5. Statistical Analysis

Continuous variables were described with mean values and standard deviation. Comparison between the dosimetric parameters obtained from dose–volume histograms of the two plans (FB and vDIBH) was performed by means of the non-parametric Mann–Whitney’s test (*p*-value < 0.05 was set as significant). We identified a LAD maximum dose ≤ 10 Gy and a LAD mean dose ≤ 4 Gy as the clinical goal. In fact, most patients (except for the upper quartile) treated with the DIBH technique received doses lower than this. We evaluated which anatomical variables were predictors of a LAD dose higher than 10 Gy (maximum dose) and 4 Gy (mean dose). Receiving operating characteristics (ROC analysis) were used to identify the cut-off point of parameters to predict the LAD maximum dose > 10 Gy and LAD mean dose > 4 Gy, and the areas under the curve (AUCs) were computed. Variables that were significantly correlated to the clinical goals (LAD maximum dose ≤ 10 Gy and a LAD mean dose ≤ 4 Gy) were included in the post-test probability computation. Post-test probability has been performed to evaluate the effect of parameter combinations [18]. Statistical analysis was carried out with the Med-Calc 11.6.1.0 statistical package (MedCalc Software, Mariakerke, Belgium).

## 3. Results

One hundred ninety-seven patients were identified, and data from 394 treatment plans were extracted and analyzed. Data were extracted from the same patients studied in FB and DIBH.

**Figure 1:**
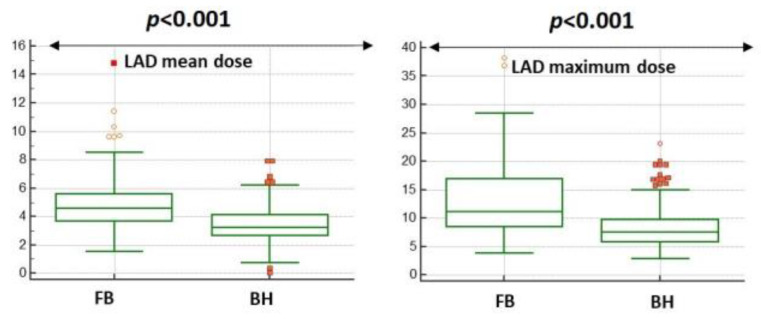
Heart dosimetry comparison between free-breathing and breath hold plans.

The LAD dose either mean or maximum was significantly reduced in DIBH plans (see Figure 1). 

In particular, the mean and maximum dose to the LAD were reduced by 31.7% (mean value 3.5 Gy vs. 4.8 Gy, *p* ≤ 0.001) and 28.1% (mean value 8.2 Gy vs. 12.8 Gy, *p* ≤ 0.001), respectively, in DIBH plans compared to FB plans. The median, mean, and interquartile range values of the mean and maximum LAD dose of both plans are summarized in Table 1. 

Several anatomic variables measured have been independently tested to predict for a LAD maximum dose > 10 Gy and for a LAD mean dose > 4 Gy. The anatomic parameters which were significantly correlated to the two clinical goals (LAD maximum dose > 10 Gy and LAD mean dose > 4 Gy) were: minimum distance from LAD to tangent open fields, lung volume and heart volume for LAD maximum dose > 10 Gy; minimum distance from LAD to tangent open fields, heart volume, lung volume, and breast separation for LAD mean dose > 4 Gy. Table 2 summarizes the AUCs for each variable cut point (only AUCs of variables with a *p*-value < 0.1 are shown) and 95% IC.

A model was built by means of post-probability test to predict the LAD maximum dose >10 Gy adding consecutively the heart volume (>655.5 cc) and lung volume (<1087.7 cc) to a minimum distance of the LAD from the tangent field (<0.1 cm). The positive predictive value (PPV) was increased from 73% to 91% (see Table 3). 

Another model was built to predict a LAD mean dose >4 Gy adding consecutively the heart volume (>652.6 cc), lung volume (>1190.6 cc), and breast separation >16.1 cm to the minimum distance of the LAD from the tangent field (<0.5 cm). The PPV was increased from 79% to 98% (see Table 3).

## 4. Discussion

The aim of this study was to analyze, in a large cohort of patients studied both in FB and DIBH, the ability of a breath-hold technique to prevent cardiotoxicity for irradiated left breast cancer. Moreover, we tried to identify anatomical and/or pre-planning characteristics correlated with the LAD dose. To the best of our knowledge, this is the largest series investigating the combination of anatomical factors in predicting LAD doses. Even if the topic is not novel, this is the first study that was able to build a model based on several FB anatomical data and capable of predicting higher doses delivered to the LAD with a PPV >90%.

Register S et al. investigated the anatomical predictors of increased heart doses among 64 women treated with tangential postoperative breast radiation therapy. He found that the main predictor of an increased MHD, volume of heart receiving 30 Gy (V30 Gy), LAD maximum dose, and volume of LAD receiving 40 Gy (LAD V40 Gy) was the heart volume included in radiation fields, which derives from treatment planning fields angles and patient’s anatomy changes occurring during breath hold. No other anatomic surrogates evaluated such as maximum heart depth, mean heart–chest wall distance, and lung breath-hold volume were found to be predictors for heart radiation exposure [14]. Similarly, Rochet et al. in a small subset of patients evaluated the role of cardiac contact distance (CCD) in guiding the physician to select patients who could benefit the most from the DIBH technique. CCD was considered as the contact of the heart silhouette with the chest wall, measured on the axial plane at the level of the dome of the diaphragm, and on a parasagittal plane at the midpoint of the left hemithorax. The study found that CCD measured on the parasagittal plane was a very good predictor for heart, LAD, and LV exposure [19]. Ferdinand et al. also reported consistent findings regarding the role of heart volume in field (HVIF) as a predictor of heart exposure. However, in this study, HVIF was correlated with all heart dosimetry parameters (maximum and mean heart dose, volume of heart receiving 5, 10, and 30 Gy) but not with the maximum LAD dose and volume of LAD receiving 5 and 40 Gy [20]. Kim et al. also combined HVIF with sternal displacement to predict the reduction in mean heart dose and mean LAD dose in 97 breast cancer patients [21].

Cao N et al. provided a very interesting prediction model for mean heart dose reduction from a set of 67 consecutive breast cancer patients. In this study, FB cardiac contact distances measured in the parasagittal plane and the FB lateral heart-to-chest distance measured in both the axial and sagittal planes were found to be the two best anatomical predictors for mean heart dose. A simple useful tool including these two parameters was provided to help clinicians in the decision process regarding the relative benefit in terms of heart exposure deriving from the use of more complex DIBH treatment techniques in individual patients [13].

In our study, the minimum distance of LAD from tangent fields was also the best anatomical variable measured in an FB CT scan able to discriminate patients at higher risk to receive a maximum LAD dose >10 Gy and a mean LAD dose of 4 Gy with a PPV of 73% and 79%, respectively. This simple and easily obtainable parameter ultimately represents the LAD portion more exposed to radiation based on treatment field geometry. Interestingly, not only negative values (LAD within the tangent fields), but also positive values (LAD close to tangent fields) were predictors of higher doses to the LAD. However, the minimum distance to the LAD alone, even if represents the best predictor of LAD exposure to radiation, is not enough to be considered. In our model, adding the other anatomic variables such as heart volume, lung volume, and/or breast separation measured on FB CT scans, we were able to increase the PPV from 73% to 91% for detecting a LAD maximum dose >10 Gy and from 79% to 98% for detecting a LAD mean dose >4 Gy. Therefore, a combination of anatomic factors adds value to the model and helps us to better understand how LAD exposure can be influenced in a multifactorial way. Therefore, all these parameters should be carefully considered.

This study has several limitations. First of all, it is a retrospective analysis. For this reason, someone can argue that measurements were collected retrospectively and, considering inter and intra-observer variability in the contouring of the LAD, this could determine unseen biases. In order to reduce this variability, all LAD contours were reviewed independently by two physicians. Moreover, in order to reduce the risk of heterogeneity in the measurement of the minimum distance of the LAD from tangent fields, this parameter was computed by the same two physicians who independently reviewed LAD contours and if the concordance was not achieved a mean value was computed.

Secondly, we chose a cut point of 10 Gy and 4 Gy for the LAD maximum and mean dose, respectively, lower than the current recommended constraints (Dmean < 10 Gy; volume of LAD receiving ≥30 Gy: <2%; volume of LAD receiving ≥40 Gy: 1%) [22]. However, we chose these dose cut-off points as 75% of patients treated with the DIBH technique in our cohort received lower doses; therefore, we believed that these cut points could be reasonable for our study purpose that aimed to underline the benefit of DIBH. Moreover, LAD radiation exposure-related damage has already been observed with such doses in clinical studies [10,11]

On the other hand, the major strength of our study relies on the large amount of data available for the analysis. In fact, we were able to propose a set of different anatomical and preplanning characteristics parameters that, combined together, increased the prediction of LAD dose exposure. This is a novel and interesting finding.

## 5. Conclusions

In conclusion, the DIBH plans achieved lower doses to the LAD in all patients and the adoption of this technique should be preferred for all patients in order to prevent cardiotoxicity, either preclinical and/or clinical; this can be particularly important taking into account that this technique suffers less from organ motion than FB and, therefore, the administered dose to the heart and its substructures is more likely to be similar to those of the treatment plan. A set of anatomic parameters combined together may accurately predict LAD exposure to radiation and therefore the relative proportion of benefit deriving from DIBH. This can be useful, especially for those patients not compliant to the DIBH technique. It can be easily used in clinical practice in order to guide selection and prevent cardiotoxicity.

## Figures and Tables

**Figure 2:**
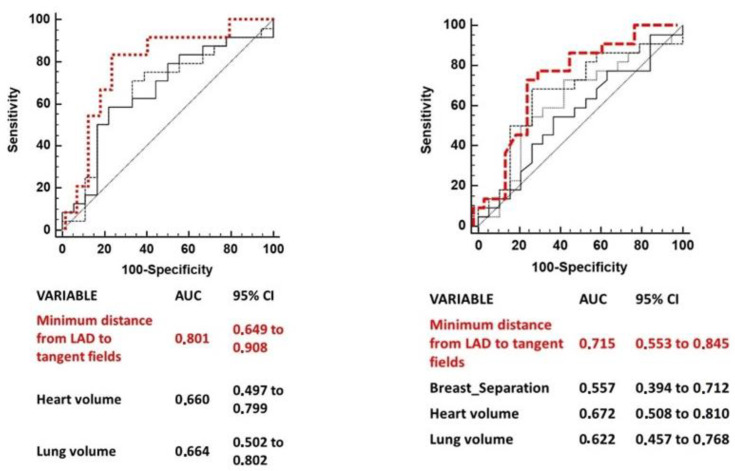
ROC curve comparison of predictors of LAD maximum dose >10 Gy (**Left**) and of LAD mean dose >4 Gy (**Right**). Legend: for left ROC curve in red minimum distance from LAD to tangent fields, medium dashed line lung volume, continuous line hearth volume; for right ROC curve in red minimum distance from LAD to tangent fields, medium dashed line hearth volume, thin dashed line lung volume, continuous line breast separation.

**Table 1:** LAD dose metrics: free-breathing (FB) and breath-hold (BH) plans, dose reduction between the FB and BH plans.

	FB Plans	BH Plans
**LAD mean dose (Gy)**		
Mean	4.8	3.5
Median	4.5	3.2
Percentiles		
25	3.6	2.6
50	4.5	3.2
75	5.5	4.1
**LAD maximum dose (Gy)**		
Mean	12.8	8.2
Median	11.1	7.5
Percentiles		
25	8.5	5.7
50	11.1	7.5
75	16.9	9.7

LAD: left descending artery.

**Table 2:** ROC analysis for predictors of the LAD Dmax > 10 Gy and LAD Dmean > 4 Gy.

LAD Dmax > 10 Gy	Cut-off Value	AUC	95%IC	*p* Value
Minimum distance from LAD to tangent fields	≤−0.11 cm	0.714	0.614–0.801	<0.0001
Lung Volume	≤1087.7 cc	0.626	0.553–0.695	0.002
Heart volume	>655.51 cc	0.660	0.497–0.799	0.07
**LAD Dmean > 4 Gy**	**Cut-off Value**	**AUC**	**95%IC**	***p* Value**
Minimum distance from LAD to tangent fields	<0.49 cm	0.687	0.588–0.744	0.001
Breast separation	>16.1 cm	0.649	0.550–0.740	0.008
Heart volume	>652.6 cc	0.672	0.508–0.810	0.051
Lung volume	≤1190.6 cc	0.624	0.519–0.722	0.050

ROC comparison curves of variables predicting a LAD maximum dose > 10 Gy and a LAD mean dose > 4 are shown in Figure 2. The minimum distance of LAD from tangent open fields was the strongest predictor of both the LAD mean and maximum dose.

**Table 3:** Predictors of the LAD Dmax > 10 and LAD Dmean > 4.

FB Scans Predictors of LAD Dmax > 10	PPV (%)
Minimum distance from LAD to tangent fields ≤−0.1 cm	73
Minimum distance from LAD to tangent fields ≤−0.1 + Heart volume >655.5 cc	87
Minimum distance from LAD to tangent fields <−0.1 + Heart Volume >655.5 cc + Lung Volume ≤1087.7	91
**FB Scans Predictors of LAD Dmean > 4**	**PPV (%)**
Minimum distance from LAD to tangent fields <0.5 cm	79
Minimum distance from LAD to tangent fields <0.5 cm + Breast separation >16.1 cm	91
Minimum distance from LAD to tangent fields <0.5 cm + Breast separation >16.1 cm + Heart volume >652.6 cc	96
Minimum distance from LAD to tangent fields <0.5 cm + Breast separation >16.1 + Heart volume >652.6 cc + Lung volume ≤1190.6	98

## Data Availability

Research data are stored in an institutional repository and will be shared upon reasonable request to the corresponding author.
